# Passively driven microfluidic device with simple operation in the development of nanolitre droplet assay in nucleic acid detection

**DOI:** 10.1038/s41598-021-00470-9

**Published:** 2021-10-25

**Authors:** Pei-Heng Lin, Bor-Ran Li

**Affiliations:** 1 Institute of Biomedical Engineering, College of Electrical and Computer Engineering, National Yang Ming Chiao Tung University, 1001 Ta-Hseh Rd., Hsinchu, Taiwan; 2 Department of Electrical and Computer Engineering, College of Electrical and Computer Engineering, National Yang Ming Chiao Tung University, Hsinchu, Taiwan; 3 Center for Emergent Functional Matter Science, National Yang Ming Chiao Tung University, Hsinchu, Taiwan

**Keywords:** Biotechnology, Chemical biology

## Abstract

Since nucleic acid amplification technology has become a vital tool for disease diagnosis, the development of precise applied nucleic acid detection technologies in point-of care testing (POCT) has become more significant. The microfluidic-based nucleic acid detection platform offers a great opportunity for on-site diagnosis efficiency, and the system is aimed at user-friendly access. Herein, we demonstrate a microfluidic system with simple operation that provides reliable nucleic acid results from 18 uniform droplets via LAMP detection. By using only micropipette regulation, users are able to control the nanoliter scale of the droplets in this valve-free and pump-free microfluidic (MF) chip. Based on the oil enclosure method and impermeable fabrication, we successfully preserved the reagent inside the microfluidic system, which significantly reduced the fluid loss and condensation. The relative standard deviation (RSD) of the fluorescence intensity between the droplets and during the heating process was < 5% and 2.0%, respectively. Additionally, for different nucleic acid detection methods, the MF-LAMP chip in this study showed good applicability to both genome detection and gene expression analysis.

## Introduction

Since polymerase chain reaction was invented in 1986, it has become one of the fundamental tools for molecular biology in medical research and clinical diagnosis^1–3^. A small amount of specific deoxyribonucleic acid (DNA) can be duplicated rapidly to generate millions of copies, providing researchers with further information for examination. Such powerful tools are often used in molecular-based diagnoses for infection identification, disease biomarker detection and cancer analysis, which are all strongly correlated with patient treatment selection and timing^4,5^. Point-of-care testing (POCT) is easily deployed in the vicinity of patients outside of the central laboratory and is recognized for providing real-time analysis results^6,7^. Due to their efficiency and convenience, POCT devices directly accelerate therapeutic intervention by capturing personal clinical information in a short amount of time^8–10^. With the increasing threat of pandemic infectious diseases through globalization, it is clear that POCT combined with molecular technologies, such as nucleic acid amplification technique, applied to frontline clinical settings can assist in early-stage implementations for public healthcare^11^. In the development of general public diagnostic applications of POCT, the ideal properties are high sensor performance and low system complexity, which potentially benefit patient management and healthcare, infectious determination, and the limitation of disease spread and ensure low resource consumption and low costs^12–17^.

The microfluidic (MF) device promises a convincing solution for nucleic acid amplification technique based on POCT diagnosis. It could be used in simple tests that obtain the results at or near patient locations over specific periods to adjust treatment in real-time. Compared to conventional amplification methods, MF-based systems are compact and portable without environmental and resource restrictions. Additionally, other functional devices, such as simple pumping systems, valves and automatic systems, can easily be combined with the microfluidic system, allowing further related analytic developments^18^. Due to several advantages, including reduced consumption of clinical sample reagents, higher reaction efficiency due to low thermal mass inertia with rapid heat transfer, portability, automation ability and reduced human operating error^19–21^, the MF system is especially of interest for nucleic acid amplification technology in the POCT field^22–24^. Many studies have incorporated nucleic acid detection devices into microfluidic systems. These integrated systems often involve active microfluidics technology, which controls fluid direction and transport with external pumps/power sources or actuators. Such a design not only increases the complexity and size of the system but also requires further human resources for operation. One of the common solutions in simplification of the microfluidic system is to provide single temperature through the nucleic acid amplification. An elimination of the temperature steps in traditional polymerase chain reaction (PCR) by circumventing the thermal heating and cooling system. This can be achieve by integrated the system with isothermal nucleic acid amplification methods, like loop-mediated isothermal amplification (LAMP), that is available for amplifying nucleic acid at a single temperature. The isothermal method substantially reduce the complexity and the power consumptions of the device^25–29^. In contrast, the challenge for simpler microfluidic system in nucleic acid testing will be how to maintain the features of the fluidic regulation, but reduce the complexity and cost of microfluidic device. Hence, “passive-driven microfluidics” technology provides a better and more advanced method that uses fluid properties and passive mechanisms to provide a better solution to meet the needs of POCT diagnosis^30,31^.

Passively driven MF avoids bulky external supporting equipment that the power source can be easily obtained in low-resource environment. Among utilizing surface tension, pressure-driven gravity, osmosis and vacuum suction^32–35^, capillary force are probably the most widely used and successful method for passive driven micropumping^36^. The flow in the microfluidic is typically drive and regulate by capillary force on the chip providing robust and easy to use advantages without any active components. Such an emerging strategy for the movement of nucleic acid samples is achieved with characteristics including easy fabrication, a lack of external power, low cost, compactness and portability. Moreover, typical nucleic acid detection users are mostly unfamiliar with MF techniques, related skills and complex operations. They are also restricted by insufficient equipment and facilities in their environment. This seriously impedes these users from adopting MF-based nucleic acid amplification devices. Passive microfluidic operation does not require professional training and experience. The straightforward operation can crucially allow the miniaturization of the overall system based on a simple operating process. In summary, the operation of the MF DNA/RNA amplification system by end-users should be as user-friendly as possible. Despite the fact that many studies claim their system only demanded pipette as auxiliary injection equipment. Most of them still require additional sealed process in the resist of water loss and bubble formation during the heating process. The sealed techniques including block inlet and outlet with tape or PDMS by O_2_ plasma treatment bonding; close the air channel by vacuum system in attempt to avoid reagent condense^37–41^. To address their easy integration of such devices, the use of a passive-driven pumping system without further additional instruments is favourable as a working principle for all users.

**Figure 1:**
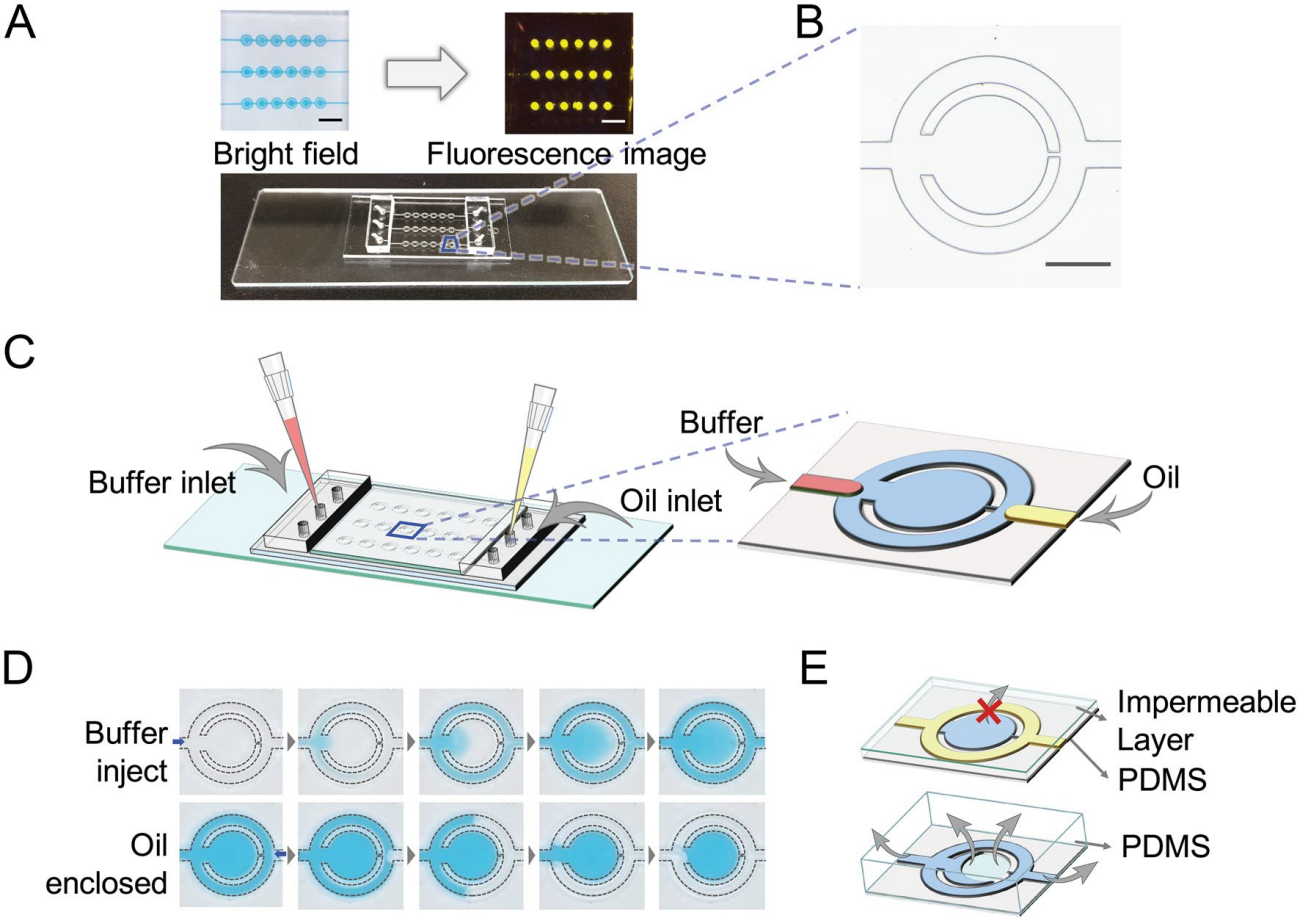
Schematic illustration of the passive-driven MF-LAMP chip. (**A**) Photograph of the MF-LAMP chip with blue dye (bright field) and fluorescence dye (fluorescence image) to indicate the microfluidic network. Scale bars are 3 mm, (**B**) Bright field image of a single chamber. Scale bars are 0.5 mm. (**C**) Simple operation of bidirectional fluid regulation by using micropipettes integrated into the MF-LAMP device. Illustration of mechanism is visualized by PowerPoint. (**D**) The process of buffer exchanging and oil enclosed method through capillary channel. (**E**) Principle of the oil enclosed and impermeable layer that prevents LAMP reagent loss by liquid/gas diffusion through the inlet and PDMS. Illustration of mechanism is visualized by PowerPoint.

In this study, we demonstrate a simple operational LAMP (loop-mediated isothermal amplification) (setup shown in Fig. 1 and Fig. S1) assay that contains uniform droplets, each of which controlled by a capillary channel for the fluid exchange and oil enclosed. Only passive auxiliaries such as pipette and syringe are needed in MF-LAMP device for reagent injection and oil can be load into the channel in order to isolate each droplet uniformly. The sealing process successfully avoid water loss and bubble formation. We also demonstrate the feasibility and stability of the MF-LAMP device through DNA/RNA LAMP assay amplification under fluorescence surveillance.

## Results and discussion

### Design concept for the simple operation MF-LAMP chip

In the presented device, the system was designed, fabricated and characterized to function as a simple-use microfluidic LAMP chip. The pump-free design and the structure of the MF-LAMP chip are shown in Fig. 1A. An MF-LAMP chip contains 3 channels each with 6 concentric circular chambers in a row. With easy operation, buffers and reagents can be effortlessly accessed and exchanged in the microfluidic channel manually with micropipettes. After reagent injection and oil enclosure, the MF-LAMP chip can be monitored in real-time during the polymerase chain reaction under a fluorescence microscope.

Figure 1B shows the image of a single microfluidic chamber. The concentric circular chamber contains a reaction chamber, a capillary channel and a peripheral channel. The reaction chamber contained approximately 60 nL of reagent in the middle for LAMP observation. Bubble interference is a critical problem, especially in MF systems, but it seems unlikely to influence the stability of the LAMP under a macroscale environment. It can severely affect a microfluidic system, however. Once bubbles form, the air trapped in the reaction chamber expands during the heating process in the LAMP. In addition, the evaporation of the reagent is also a serious problem that leads to changes in both the concentration and volume in large surface-to-volume ratio microfluidic systems.

With the control and regulation of fluid access and preservation, the mechanism is ensured through the geometric structure of the microfluidic design. The basic principle of MF-LAMP operation is illustrated in Fig. 1C. For fluid injection, fluid enters the channel through the buffer inlet, compressing the air equally throughout the entire channel. Additionally, the solution for fluid exchange during the experiment is injected through the buffer inlet. In the enclosed method, as the oil travels through the microfluidic device, the oil stops in front of the capillary channel and bypasses the peripheral channel, causing the remaining reagents to be trapped in the reaction chambers (Fig. 1D and Movie S1). The micropipette as a manual handling tool does not lead to a large error in the droplet volume since the structure is preset in the designed microfluidic system. Hence, bidirectional MF-LAMP is effectively implemented to generate LAMP droplets, avoiding fluid evaporation by simply using micropipettes for operation.

To avoid reagent loss during LAMP thermal heating, oil is applied to the peripheral channel that surrounds the reaction chambers, protecting the middle of the chamber from buffer evaporation. Although the oil enclosure protects the stability of the reagent, the material of the microchannel structure can allow gas dissipation. PDMS, a widely used polymer in microfluidic fabrication, triggers massive gas escape during the heating process. Thin films such as Parylene C, fluorosilane polymer and polyethylene as gas vapor barriers are integrated into the MF chip^42–44^. However, the process of depositing these materials is complex and time-consuming. In this study, we used a glass slide as the impermeable layer attached to the thin layer of the PDMS microfluidic channel. The impermeable layer binds to the PDMS surface easily directly on the top of the reaction area, avoiding the supporting layer after 1 min of oxygen plasma treatment. In the presence of the impermeable layer and the enclosed oil, the reagent was well preserved in the reaction chamber during the heating process of the LAMP (Fig. 1E). In the absence of both of these features, the molecules inside the reaction chamber evaporate quickly, generating bubbles and condensing the reagent, which severely interferes with the LAMP. In addition, due to their easy access and low cost, glass slides can effectively prevent reduction of the system stability.

### Principle of capillary channel control

**Figure 2:**
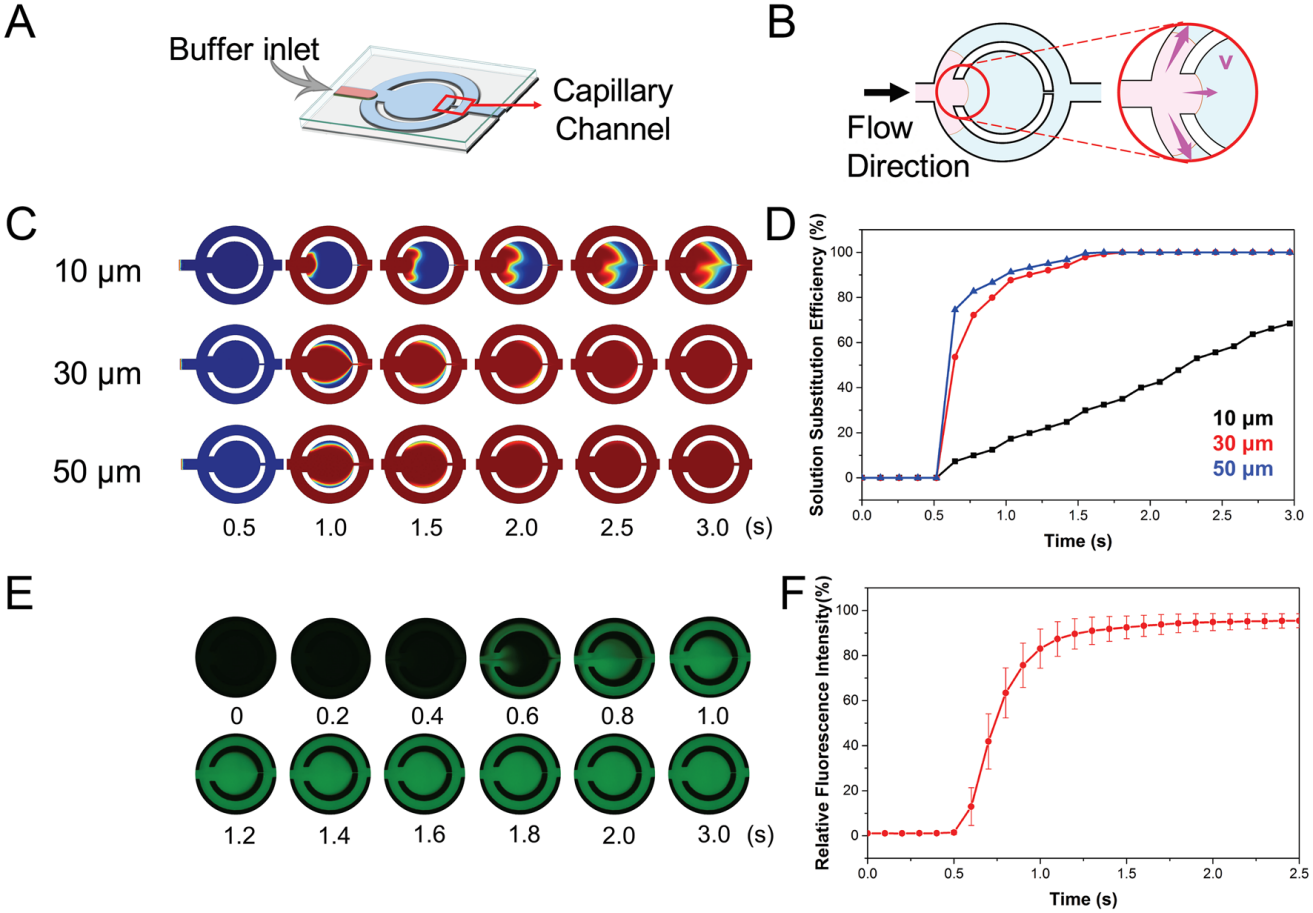
Fluid substitution performance of the MF-LAMP chip. (**A**,**B**) Schematic of the buffer inlet direction of the MF-LAMP chip. Illustration of mechanism is visualized by PowerPoint. (**C**) Illustration of the computational simulation domain in a single chamber with various capillary channel widths (10, 30 and 50 µm) visualized by COMSOL. (**D**) Comparison of the fluid substitution efficiency in the simulation result. (**E**) Time-sequence snapshots of the dynamics of dilution by water injection in the fluorescence droplet and the relative intensity variance (**F**) throughout the injection process.

The capillary channel is a crucial element of the regulation of the passive-driven MF-LAMP device for fluid retainment or passage, as shown in Fig. 2A. The width of the capillary channel directly affects the solution substitution and oil enclosure efficiency in this system. The preparation process requires buffer washing and reagent displacement from the buffer inlet, and the solution exchange efficiency is strongly dependent on the width of the capillary channel (Fig. 2B). The narrow capillary channel limits the fluid velocity in the reaction chamber, resulting in a low exchange efficiency for the buffer. In the simulation of the buffer exchange efficiency for capillary channels of different widths, the flow characteristics were solved by using the simplified Navier-Stoke equation and continuity equation:

1 $$0=\nabla \cdot[-\rho {\varvec{l}}+\mu \left(\nabla {\varvec{u}}+{\left(\nabla {\varvec{u}}\right)}^{\mathrm{T}}\right]+F$$

2 $$\rho \nabla \cdot \left({\varvec{u}}\right)=0$$

where **u** is the fluid velocity, ∇ represents the gradient, p is the fluid pressure, ρ is the fluid density, μ is the fluid dynamic viscosity, F is the external force applied to the fluid, I is the identity matrix and T is the matrix transpose function. The wall condition was set to no slip. Additionally, the convection and diffusion effects in the mass transport for the concentration gradients were considered in the solution exchange simulation.

3 $${{\varvec{N}}}_{i}=-{D}_{i}\nabla {c}_{i}+{c}_{i}{\varvec{u}}$$

4 $$\frac{\partial {c}_{i}}{\partial t}+\nabla \cdot {{\varvec{N}}}_{i}=0$$

where **N**_i_ is the transport flux of the species, ci is the species concentration, Di is the species diffusivity and **u** is the fluid velocity.

In the initial stage (0–0.5 s), the channel is prefilled with one solution (shown in blue), while the other solution, shown in red, enters the buffer inlet subsequently at 0.5 s with a flow rate of 0.6 mL min^−1^. The mixing process is presented in Fig. 2C with channel widths of 10, 30 and 50 μm. The wider capillary channel shows a higher exchange rate with a higher velocity in the reaction chamber. The solution substitution ratio for both the 30 and 50 μm channel widths reached 100% within 2.5 s. In contrast, the 10 μm channel requires a longer substitution time due to the slow fluid velocity in the reaction chamber, which leads to increased reagent consumption. The solution substitution efficiency demonstrated that within 1 s, the 30- and 50-μm channels achieved 100% solution replacement. However, the narrow-width 10 μm channel requires a longer time (> 2.5 s) for solution substitution (Fig. 2D). To better clarify the velocity distribution, the velocity profiles across the Y-axis in the reaction chamber are shown in Fig. S2.

Compared to the peripheral area, the central areas of the reaction chamber showed a higher velocity that corresponded to the solution exchange process, with the substituted solution observed first in the center and then expanding into the peripheral area. It also shows the velocity decrease with the decrease in the width of the capillary channel. To test the performance in actual operation for solution exchange, distilled water was preinjected into the reaction chamber. With a micropipette, 1 µg mL^−1^ fluorescein in distilled water was injected into the channel (Fig. 2E); the fluorescence intensity response was recorded, and the exchange efficiency was calculated. Within 2.5 s, the fluorescence solution was 100% substituted by the distilled water, which was similar to the simulation result (Fig. 2F).

**Figure 3:**
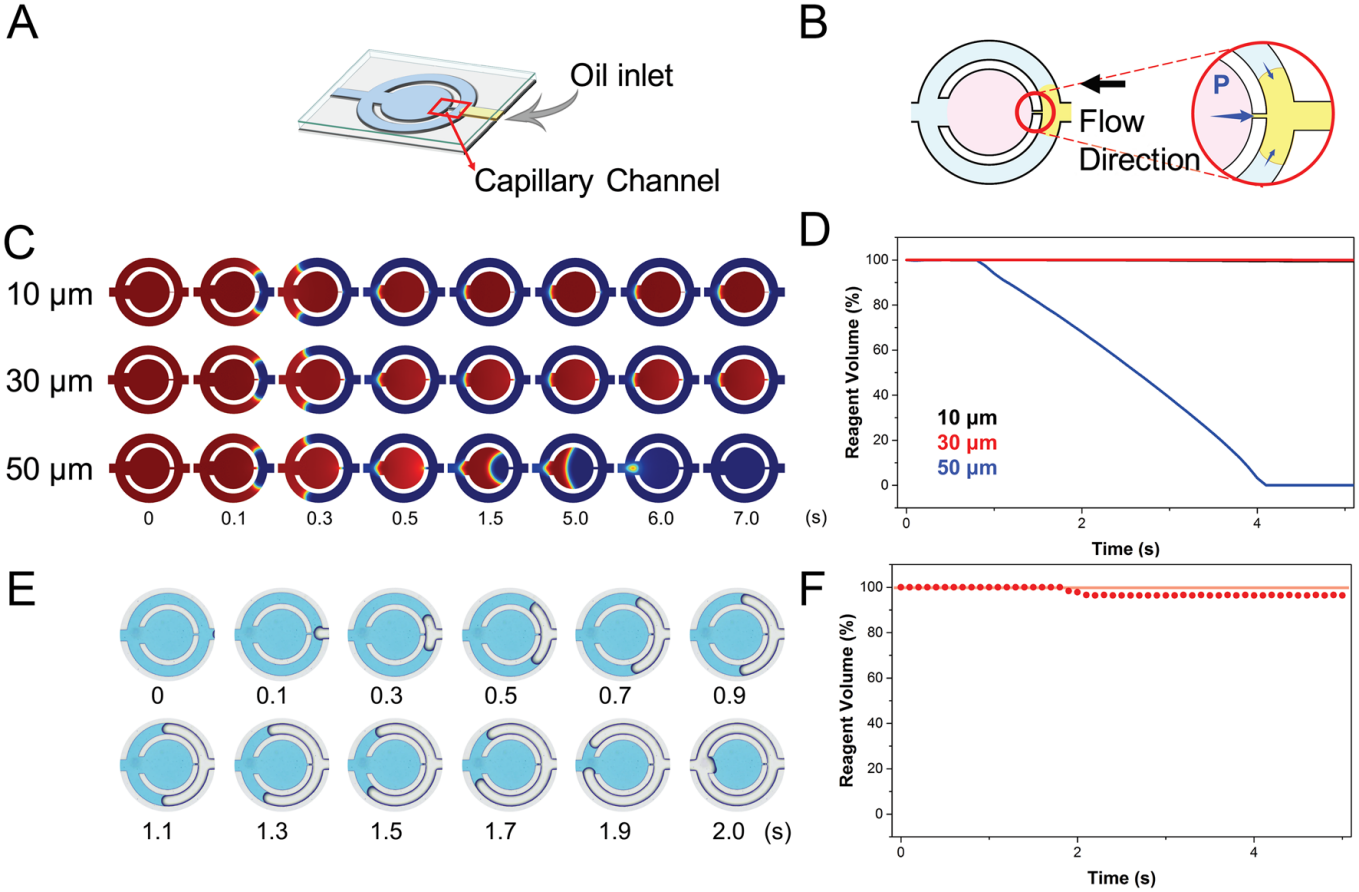
Oil enclosure performance of the MF-LAMP chip with varied capillary channels. (**A**) Schematic of the oil inlet direction of the MF-LAMP chip. (**B**) Capillary force that stopped oil insertion into the reaction chamber. Illustration of mechanism is visualized by PowerPoint. (**C**) Computational simulation of the single chamber domain through the oil enclosure process with varied channel widths (10, 30 and 50 µm) and reagent volume loss. Illustration is visualized by COMSOL (**D**). (**E**) Actual oil enclosure process with blue dye used as the LAMP reagent for visualization; the reagent volume variation is plotted in (**F**) and shown as red dots.

In the process of sample loading, formation of stationary droplets with stable volume and conditions is necessary for a LAMP droplet assay. Additionally, oil enclosure was adopted to ensure a more reliable process by decreasing the water loss in the reaction chamber during thermal heating. As the mineral oil enters the microchannel from the opposite direction of the buffer inlet (Fig. 3A), it travels along the microfluidic system and is stopped by the capillary channel. This capillary barrier is achieved by abrupt expansion of different sections of the wettable microchannels that break up the aqueous reagent, generating uniform droplets depending on the microfluidic geometry^45^. The Young–Laplace equation describes the bypass pressure in a rectangular microchannel as

5 $${P}_{A}-{P}_{B}=-2\sigma \left(\frac{cos{\theta }_{s}}{w}+\frac{cos{\theta }_{v}}{h}\right)$$

where w and h are the width and height of the microchannel, and σ represents the surface tension of the liquid. The advancing contact angle of the sidewall is θ_s_, and that of the top and bottom is θ_v_. The pressure barrier of the fluid expansion in this case depends on the geometry of the channel, while the surface tension and contact angles remain the same. By the time the oil reached the capillary channel, as long as the insertion pressure did not exceed the bursting pressure, the oil tended to bypass the reaction chamber and flow through the outer channel (Fig. 3B), where it was divided into uniform droplets with oil protection. As determined by the numerical simulation, Fig. 3C illustrates the simulated domain with different capillary channel widths (10, 30 and 50 μm). The contact angle of the oil with PDMS in the aqueous phase was set as 120°. During injection at 5 mm s^−1^, the advancing oil was stopped at the 10- and 30-μm capillary channels, preserving the LAMP reagents in the reaction chamber. In contrast, the 50 μm capillary channel had a lower bursting pressure, which was the result of increasing the width in Eq. (5), and the insertion pressure easily reached the threshold value. The simulated illustration shows that the fluid in the reaction chamber was pushed out during the oil enclosure, with reagent loss (Fig. 3D). The actual oil loading process is shown in Fig. 3E, and blue dye was used to provide better visualization of the oil enclosed process. The reagent volume variation is calculated by the image j of the ratio of V_oil_ and V_i_, where the initial volume in the central reaction chamber is V_i_ and the reagent volume after oil injection is V_oil_. The reagent was 100% in volume, which remained in the reaction chamber during the following reaction and observation (Fig. 3F). Oil insertion not only divided reagents into uniform droplets but also prevented evaporation during the heating process.

Based on the simulation and actual testing results, we suggest the proper design of the geometry of the MF-LAMP chamber. Despite the fact that the narrow width is more appropriate for oil-enclosed regulation of the bursting pressure threshold, the velocity in the reaction chamber is quite low, resulting in low solution substitution efficiency.

### Characterization of the MF-LAMP device

**Figure 4:**
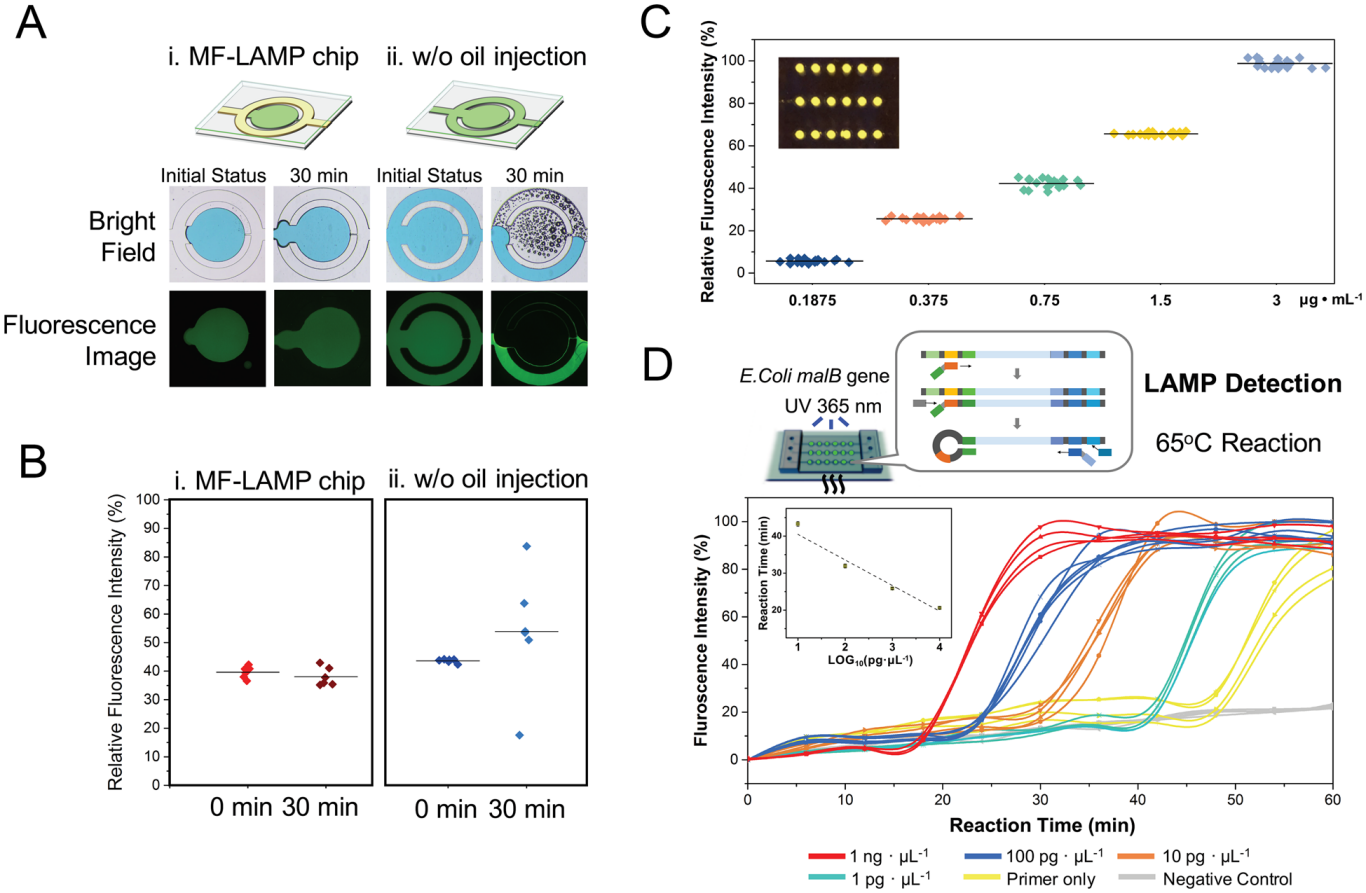
Application of the MF-LAMP chip. (**A**) Different fabrication strategies of MF-LAMP with or without enclosed oil. The colored ink and fluorescence dye allow better observation of the variation of different permeable methods after the heating process. Illustration is visualized by PowerPoint. (**B**) Comparison of each method according to fluorescence variance between the initial status and the after heating status. (**C**) Analysis of the uniformity of the intensity in each reaction chamber with different fluorescence concentrations. (**D**) Amplification curves for detecting different concentrations of *E. coli* DNA (10^3^, 10^2^, 10, and 1 pg µL^−1^) generated by the processing of the fluorescence images. Illustration of mechanism is visualized by PowerPoint.

Maintaining the proper concentration is vital for quantification LAMP method. The slight differences in samples, primers, nucleotides, ions, buffers and temperature strongly affect the function and efficiency of polymerase replication. To test the stability of the reagent, fluorescent dye was injected into the reaction chamber with enclosed oil. Figure 4A illustrates the different chip methods, including the MF-LAMP method (with oil enclosure) and enclosed heating method without oil at 70 °C for 30 min. The initial and after heating images depict the effect of different strategies of chip fabrication, and blue dye and fluorescent dye were inserted into the microfluidic device for bright field microscopy and fluorescence image acquisition, respectively. The variation between the initial status and the status after heating for 30 min for the fluorescence intensity is shown in Fig. 4B. In the MF-LAMP chip, the solution maintained a well-preserved condition with no bubble interference in the reaction. The relative standard deviation was measured as 1.8% in the initial state and 4.8% after 30 min of the heating process.

The outer peripheral channel could also be the buffer area for liquid expansion during increases in heat. In contrast, the chip without enclosed oil showed great variation after the heating process. Without oil protection, evaporation easily occurs in the inlet adjacent channel, with massive water loss. Bubbles also invaded both ends of the reaction chamber and even squeezed out the reagent inside the chamber, resulting in difficulties in fluorescence detection. Since there was no oil to divide the reagent into uniform droplets, the evaporation also led to condensation of the reagent in the middle area, resulting in high fluorescence intensity and great variation in the RSD value (1.4% in the initial state and 36.4% after thermal heating). Additionally, in the fabrication of the MF-LAMP chip, we also considered the gas/liquid permeability of PDMS during the heating process.

The functional, impermeable layer was adherent to the high porosity PDMS as a vertical gas/liquid barrier that lowered the diffusion of solution. In comparison to the necessity of each functional process or layer, different methods, including the MF-LAMP method (with both oil enclosure and the impermeable layer) and methods without oil enclosure or an impermeable layer after heating at 70 °C for 30 min, are shown in Fig. S3. With high porosity, the PDMS polymer provides “openings”, allowing gas molecules to diffuse inside the networks. Especially under thermal heating conditions, the transport of gas/liquid molecules through PDMS increases due to the high pressure gradient between the reaction chamber and the external environment. Such an increased mass flow rate generates water dissipation and condenses the reagent inside the reaction chamber, resulting in high fluorescent intensity both in the fluorescence images and the trend graph. The RSD over time for MF-LAMP is 2.0%, which is relatively lower than that observed without oil enclosure (10.1%) and without an impermeable layer (11.5%). Consequently, no bubble invasion or reagent loss occurred in the MF-LAMP chip during thermal heating. Both results showed that our approaches for MF-LAMP chips are effective in suppressing bubble formation and solution condensation.

### Application of the MF-LAMP device for diagnosis

For the MF-LAMP assay, the droplet assay was performed under oil with a uniform composition depending on the geometric structure of the microfluidic design. To evaluate the performance of droplet manipulation under manual operation conditions, we generated a 3 × 6 assay of a single droplet filled with various fluorescence dye concentrations of 0.1875, 0.375, 0.75, 1.5 and 3 μg mL^−1^ (Fig. 4C). The fluorescence intensities in each assay were measured, and the relative standard deviations (RSDs) were calculated as 15.6%, 3.2%, 5.0%, 1.0% and 2.0% (n = 18). Under a low fluorescence concentration, the measured RSD was relatively high, and the weak intensity was easily affected by the background noise. On the other hand, in the range from 0.375 to 3 μg mL^−1^, a low RSD resulted, which showed the high precision and reliability of the MF-LAMP device with uniform droplet manipulation under manual operation mode.

To evaluate the usability and sensitivity of the MF-LAMP device for nucleic acid amplification application, *Escherichia coli* (*E. coli*) *malB* gene samples with a wide range of concentrations (from 1 ng μL^−1^ to 1 pg μL^−1^) in the droplet assay were measured. The target samples were mixed with LAMP primers and reagents and inserted into the MF-LAMP chip, followed by oil enclosure for channel sealing for the heating process. The fluorescent images of droplets were recorded, and the corresponding real-time intensity variation curves are presented in Fig. 4D as the linear fitted graph of the logarithm of the concentration of *malB* template versus the reaction time, and the linear relationship was obtained (R^2^ = 0.94). The standard deviations of the reaction times were 0.645, 0.545, 0.288 and 0.328 for concentrations of 1 ng μL^−1^, 100 pg μL^−1^, 10 pg μL^−1^ and 1 pg μL^−1^ (n = 6), respectively. The gel electrophoresis after reaction was showed in Fig. S4. The result showed that there is difference in 1 pg μL^−1^ and primer only solution. The proper incubation time should adjust to 30 to 40 min by adjustment of the copies/ml in the clinical sample. For this reason, we adjust the LAMP reaction time within 60 min and suggesting the proper quantification time around 30 to 40 min. It makes sure that the signal amplification that they are seeing is not coming from primer dimerization. In addition to gene detection, we also demonstrated the mRNA expression of the mutant epidermal growth factor receptor (EGFR) gene in H1975 cell lines on the MF-LAMP chip, and the results are shown in Fig. S5. These results demonstrate the uniformity and feasibility of the droplet assay for both gene detection and mRNA expression detection by using the nucleic acid amplification microfluidic technique under manual operation with simple steps. Additionally, because of its high sensitivity of detection, MF-LAMP also substantially reduces sample and reagent consumption.

## Conclusion

In summary, we demonstrate a simple operation microfluidic diagnosis system that provides reliable nucleic acid analysis results. No external equipment was required to pump or to control the flow and droplet formation. Compared to other studies, the simple principle only requires regular manual micropipettes without additional assisted sealing equipment (Table 1). Carefully designed structural channel and capillary valve held the incoming reagent in place and partitioning uniform droplet of 60 nL volume for each. This passive-driven device regulates the nanoliter scale of droplets, significantly reducing both the reagent consumption and operation steps. The oil enclosure method and impermeable layer create a gas/liquid barrier that minimizes the solution evaporation and bubble formation, which greatly maintains the reagent stability (RSD = 4.8% in each chamber and 2.0% over time). Our approach was successfully used for both gene and RNA expression detection, indicating the flexibility of the MF-LAMP device. This MF-LAMP chip showed stable quantitative performance with a high sensitivity of 1 pg μL^−1^ for the *E. coli mal B* gene.

**Table 1:** Comparison of recent passive-driven approach of nucleic acid amplification microfluidic device.

Technology	Amplification technique	Material used	Sealed method	Auxiliaries involved	Characteristic of assay	References
Centrifugal Microfluidic Platform	PCR	Polymethyl methacrylate (PMMA)	Mineral oil injection	Rotary motor	HBV detection from whole blood based on centrifugal microfluidic platform. Pipette injection with mineral oil	^35^
Smartphone-based multiplex test	LAMP	Sillicon wafer with SiO_2_ film as microfluidic channel	Sealed with double side adhesive layer and covered with glass on top	Pipette	Fluorescence images of LAMP amplification reaction were taken by a smartphone	^37^
SlipChip/ sp-SlipChip	LAMP	Two layer of glass plate	Lubricating oil were placed between two plates	Pipette	Reagent self-partition into individual droplets driven by surface tension-driven with slipping the two plate	^38,39^
Self-driven microfluidic chip	LAMP	PDMS with hydrophilic UV cured glue	Closed the channel with air-control channel via vacuum system	Pipette, electro-magnetic valves, compact vacuum pump	Hydrophilic surface drives the LAMP reagent flow into the channel	^40^
MF-LAMP	LAMP	PDMS, Glass	Mineral oil injection and outer channel surrounded	Pipette	Capillary channel controls the fluid exchange, partition, sealed process avoiding water loss in the device	This study

The development of the rapid and user-friendly MF device that cooperated with isothermal LAMP method holds a promising and robust nucleic acid detections for bacteria, virus and gene in biological samples. It definitely lower the complexity and power consumption of the system and makes the LAMP-MF system appropriate in the revolution of point-of care diagnostic. As an outlook, the MF-LAMP has the great potential in systematically integrated with the extraction process of the nucleic acid, that include pre- encapsulated extraction amplification reagent in to the device. This approach would help spread the use in POCT applications and clinical diagnosis in the low-resource settings but encounter big challenge in the process of realization. The primary component in the device like LAMP extraction reagent requires to maintain in the cold temperature. This limited the device application range especially in a low resource environment with lack of preservation equipment. With assistance from the more stable reagent, an inexpensive and portable device is suitable for the urgent need to develop rapid and simple POCT for highly contagious diseases such as SARS-CoV-2 in the low resource environment. Our method provides for simple operation for rapid nucleic acid detection in a varied diagnosis field that offers instant results for patients and physicians and can generally enhance diagnostic ability for regional disease surveillance.

## Materials and methods

### Materials and equipment

Chemical reagents, including mineral oil (light), NaCl, KCl, Na_2_HPO_4_, KH_2_PO_4_ and Tween 20, were purchased from Sigma Aldrich (Missouri, USA). Loop-mediated isothermal amplification (LAMP) kits (RNA/DNA Amplification Reagent D), fluorescein, polydimethyl siloxane (PDMS), BSA (bovine serum albumin) and glass slide were purchased from EIKEN (Tokyo, Japan), Assemzyme (Taipei, Taiwan), Silmore (Taipei, Taiwan), Chumeia (Hsinchu, Taiwan) and Protech (Taipei, Taiwan), respectively. The instruments, including the heating platform and the O_2_ plasma bonding device (Zepto Plasma Cleaner), were purchased from Yscco, Hsinchu, Taiwan, and Diener, Bielefeld, Germany, respectively. Inverted fluorescent LS620 microscope (Etaluma, Carlsbad, CA) with a CCD camera and LED light source and upright microscope (Dino-Lite digital LED microscope, AnMo Electronics Corporation, Taiwan, Taipei) was used in this study.

### Fabrication of the microfluidic LAMP chip

The MF-LAMP chip was fabricated with PDMS with the silicon wafer etching lithographic technique. Briefly, the MF-LAMP device contained a total of 18 reaction chambers. The diameter and height of each reaction chamber were 450 μm and 100 μm, respectively. The volume of the reagent droplet was approximately 60 nL. The narrow channel next to the reaction chamber was designed to be 30 μm wide. For the MF-LAMP, the width and height of the peripheral channel were set as 200 μm and 100 μm, respectively.

Polydimethyl siloxane (PDMS) and its curing agent were mixed at a 10:1 ratio and poured onto the fabricated wafer. After degassing for several minutes, the mold was heated at 80 °C for 2 h. The cured PDMS was removed from the MF mold, and an external injection hole was created by a 1.5 mm diameter puncher for the sample inlet. The microfluidic channel was trimmed for the following layer adhesion procedure.

The MF-LAMP device contained four layers, as shown in Fig. S1. The bottom layer is a glass slide with a size of 25.4 × 76.2 mm and a 1-mm thickness, which served as the fundamental chip. A microfluidic channel made of PDMS was bonded to the top of the glass slide by O_2_ plasma, ensuring that the MF channel and reaction chambers were sealed inside. Another glass slide with a size of 16.2 × 16.2 mm (1-mm thickness) was bonded to the top area of the reaction chambers, preventing gas diffusion during the heating process.

Since the previously mentioned layers were less than 1 mm thick, it is inconvenient to operate the amplification system with the sample solution. A supporting layer was added to the inlets of the microfluidic channel with a 2.6-mm thickness that allows the placement of the micropipette tip. Oxygen plasma created by a plasma cleaner was used to bond the PDMS and glass slide. The bonded PDMS chips were then placed in an oven at 90 °C for 2 h to ensure good bonding between both surfaces. The PDMS chips were placed at room temperature for at least 1 day to increase the stability of the surface wetting hydrophobicity. To avoid nonspecific adsorption, which affects the continuation of the flow and causes high background noise in the microfluidic channel, 50 mg/mL BSA in PBST was injected into the MF-LAMP channel for 30 min. After blocking with BSA, PBST buffer was injected into the channel to exchange the blocking buffer^46^.

### Fluorescence intensity testing of the MF-LAMP chip

For the solution substitution, heating and uniformity tests, water-soluble fluorescein was used as the illustration dye for visualization of the conditions in the reaction chamber in the MF-LAMP chip. To test the characteristics of the MF-LAMP chip during the heating process, fluorescein was added to the reaction chamber. The fluorescent dye was preheated to 70 °C, and the intensity was recorded after 1 min of MF heating during the thermal cycle to allow the fluorescence intensity to remain uniform. The upright microscope was used to monitored the fluorescence intensity variation while the MF-LAMP chip was isothermal heated on a hot-plate. For clear fluorescent image in individual reaction chamber, we have used inverted microscope to capture the fluorescent image with a heated transparent platform below the MF-LAMP chip in order to maintain the isothermal temperature. Both the fluorescence intensity and quantification LAMP images were analyzed by ImageJ software.

### Bacterial genomic DNA and cellular RNA extraction

Genomic DNA from *Escherichia coli* was obtained from the Culture Collection and Research Center (Food Industry Research and Development Institute, HsinChu, Taiwan) and cultivated in Luria–Bertani (LB) broth in an incubator for 16 h at 37 °C. Genomic DNA extraction was performed at 95 °C for 5 min to denature the proteins. The precise concentrations of genomic DNA and mRNA were measured by a microvolume spectrometer (Titertek-Berthold, Pforzheim, Germany).

### LAMP quantification

In this study, the loop-mediated isothermal amplification (LAMP) method was applied to the MF-LAMP device. DNA and RNA could be amplified to generate 10^9^ copies of the target sequence in less than one hour under a constant temperature (60–65 °C). Although the primer design is more complex than that used for general PCR, the process of LAMP is considered to be easier to use for unprocessed sample operations and less susceptible to the existence of PCR inhibitors^47,48^. LAMP has been used for POCT applications in nucleic acid testing, which evidently reduces the complexity of the process operation, and was applied in this study. LAMP assays were designed for specific nucleic acid sequences for detection of genomic DNA and RNA expression. Genomic DNA was used to detect *E. coli* species by recognizing a conserved region (*E. coli malB* gene)^49^. For mRNA expression, we chose the L858R point mutation in exon 21 of the epidermal growth factor receptor (EGFR) gene^50^. The primer sets are presented in Table S1.

To prepare the LAMP assay, 15 μL primer mix (40 pmol FIP and BIP, 20 pmol LF and LB, and 5 pmol F3 and B3) and 10 μL of sample solution were added into the reaction tube, which contained dry-formulated nucleic acid (RNA/DNA amplification reagent). After inversion of the tube 5 times and centrifugation of the reagent, the LAMP mixture was added into the MF-LAMP chip used in this study. Mineral oil was added into the channel through the oil inlet for reagent enclosure. The device was incubated at 65 °C for 60 min under fluorescence monitoring.

### Theoretical principle of the MF-LAMP device

In the process of buffer washing, sample loading and oil enclosure, the width of the capillary channel controls the mixing efficiency and oil enclosure efficiency inside the reaction chamber. To analyze the behavior in the reaction chamber, we used the COMSOL Multiphysics software to perform the modeling (COMSOL Multiphysics 5.5; Comsol, Inc.) COMSOL is bought via local agent (Pitotech, Changhua, Taiwan) with License No: 5087629 Windows/Mac. In the simulation, the analyzed domain was simplified to be 2 dimensional and to consist of only a single LAMP chamber. The constructed CFD model was used as the same structure in the actual model, with only a change of the capillary channel width to 10, 30 or 50 μm. The fluid is assumed to be laminar and incompressible Newtonian fluid. The temperature in this study was set to 25 °C.

## Supplementary Information

Supplementary Video 1.

Supplementary Information 1.
