# Supplementary material for: Passively driven microfluidic device with simple operation in the development of nanolitre droplet assay in nucleic acid detection
Source: Sci Rep. 2021 Oct 25;11:21019. doi: 10.1038/s41598-021-00470-9 (PMC8549005; doi:10.1038/s41598-021-00470-9)
Supplement: Supplementary file 2 — Supplementary Information 1. [file 41598_2021_470_MOESM2_ESM.pdf]

Passively driven microfluidic device with simple operation in the development of nanolitre droplet assay in nucleic acid detection

Pei-Heng Lin[1, 2] & Bor-Ran Li[1,2,3*]

1. Institute of Biomedical Engineering, College of Electrical and Computer Engineering, National Yang Ming Chiao Tung University, Hsinchu, Taiwan.
2. Department of Electrical and Computer Engineering, College of Electrical and Computer Engineering, National Yang Ming Chiao Tung University, Hsinchu, Taiwan.
3. Center for Emergent Functional Matter Science, National Yang Ming Chiao Tung University, Hsinchu, Taiwan

* To whom correspondence should be addressed.

Dr. Bor-Ran Li
Address: Institute of Biomedical Engineering, National Yang Ming Chiao Tung University, 1001 Ta-Hseh Rd. Hsinchu, Taiwan
Tel No: 886-3-5712121 ext. 54051
Fax No: 886-3-5165993
E-mail: liborran@nycu.edu.tw

Table S1. The LAMP designed primer sets

E.coli. malB gene	
F3	GCCATCTCCTGATGACGC
B3	ATTTACCGCAGCCAGACG
FIP	CTGGGGCGAGGTCGTGGTATTCCGACAAACACCACGAATT
BIP	CATTTTGCAGCTGTACGCTCGCAGCCCATCATGAATGTTGCT
LF	CTTTGTAACAACCTGTCATCGACA
LB	ATCAATCTCGATATCCATGAAGGTG
H1975 cell lines mutant EGFR L8585 expression	
F3	GCAGGGTCTTCTCTGTTTCA
B3	TCAGGAAAATGCTGGCTGAC
FIP	TTCACCAGTACGTTCCTGGCTGAACTACTTGGAGGACCGTCG
BIP	GTGCCAAACTGCTGGGTGCAGCCACCTCCTTACTTTGC
LF	AGGTCGCGGTGCACCAAG
LB	AAGAGAAAGAATACCATGCAGAAGG

Movie S1. The video of MF-LAMP operation. The oil stops in front of the capillary channel and bypasses the peripheral channel, causing the remaining reagents to be trapped in the reaction chambers.

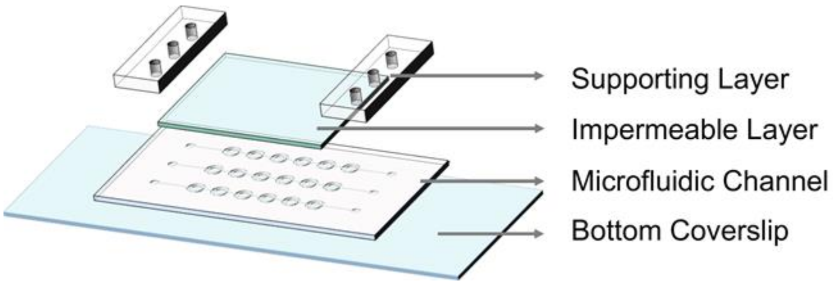

Fig. S1. Schematic illustration of each layer of the MF-LAMP chip with different functions for reliable nucleic acid detection.

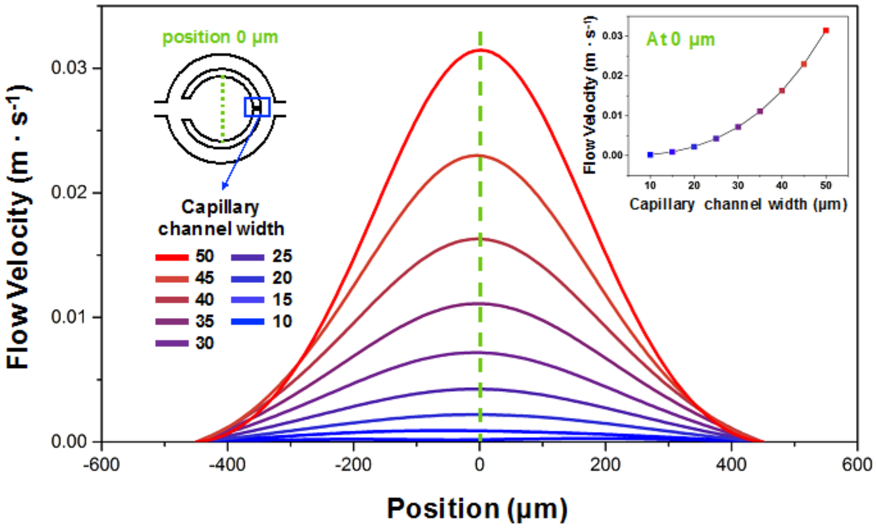

Fig. S2. Computational simulation result for fluid substitution. The flow velocity distribution in the reaction chamber with a varied capillary channel width. The velocity in the center (at 0 μm) is shown in the upper right corner.

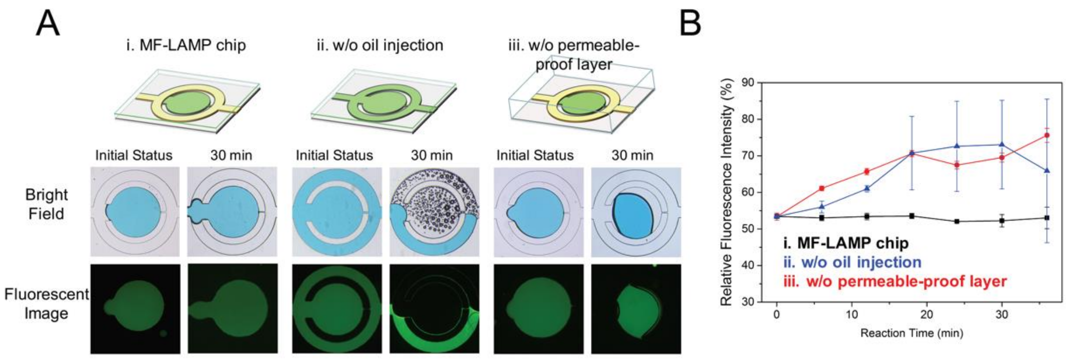

Fig. S3. Characteristics of the MF-PCR chip with different fabrication methods during the heating process. (A) shows different fabrication strategies for the MF-LAMP, the method without oil injection and the method without an impermeable layer with heating at 70°C for 30 minutes. Colored dye and fluorescence dye were used for clarity. (B) Variation in fluorescence intensity over time.

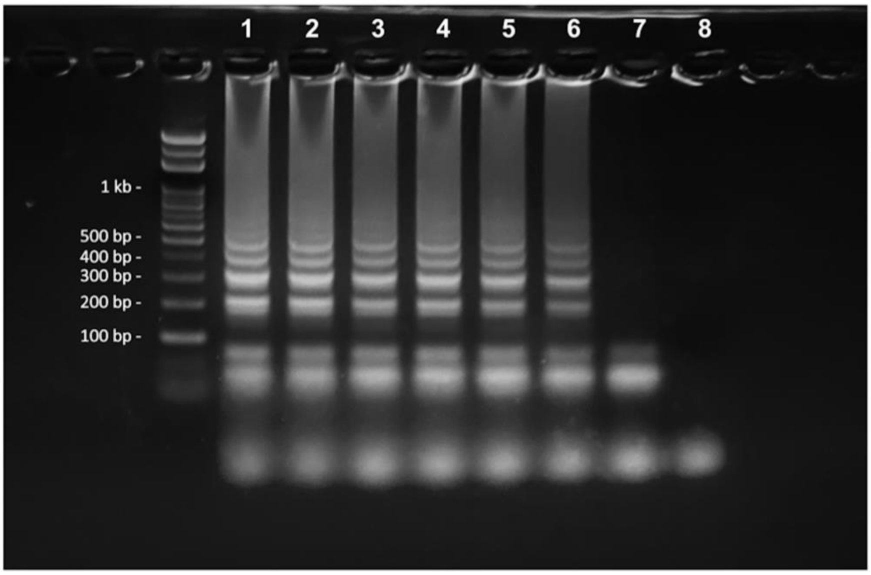

Fig. S4. The gel electrophoresis after reaction. lane (1) 100 ng.uL^{-1} (2) 10 ng .uL^{-1} (3) 1 ng .uL^{-1} (4) 100 pg .uL^{-1} (5) 10 pg .uL^{-1} (6) 1 pg .uL^{-1} (7) primer only (8) negative control.

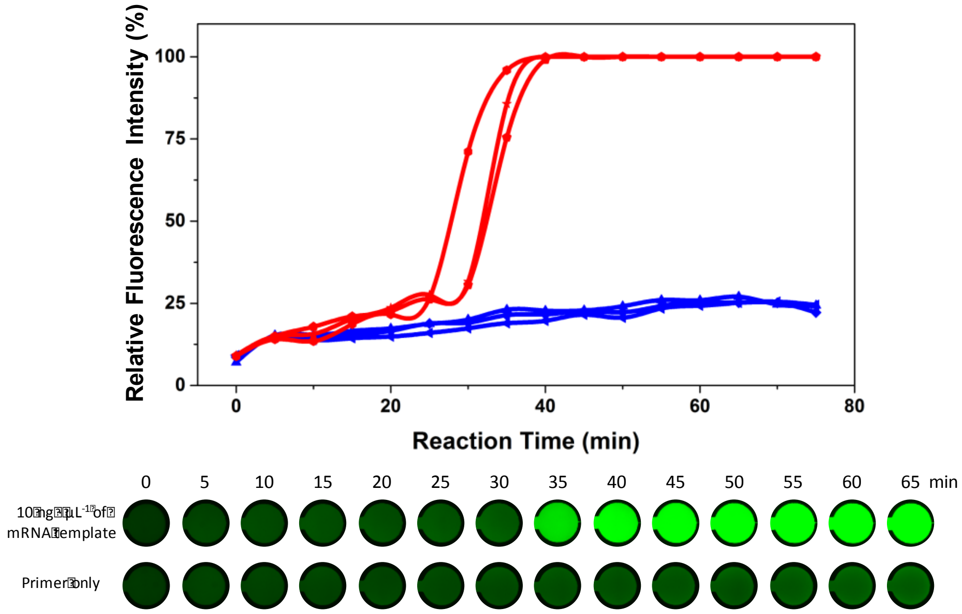

Fig. S5. Amplification graph and image of mutant EGFR expression in the H1975 cell line. Ten nanograms of mRNA template (red line) resulted in a threshold cycle of approximately 30 minutes, and the negative control (primer only, without template; blue line) showed no significant intensity change. The fluorescence images obtained during the reaction are shown below.
